# Main differential endometrial microbiota associated with recurrent implantation failure: a case control study

**DOI:** 10.3389/fendo.2025.1504609

**Published:** 2025-08-11

**Authors:** Hong Gao, Jing Xiao, Bingyan Liang, Xiaolan Wang, Huanhuan Li, Genlin Li, Baoyu Wu

**Affiliations:** ^1^ Department of Nursing, The Second Affiliated Hospital, Hengyang Medical School, University of South China, Hengyang, Hunan, China; ^2^ Ottawa Hospital Research Institute, The Ottawa Hospital, Ottawa, ON, Canada; ^3^ School of Nursing, University of South China, Hengyang, Hunan, China; ^4^ Center for a Combination of Obstetrics and Gynecology & Reproductive Medicine, The First Affiliated Hospital, Hengyang Medical School, University of South China, Hengyang, Hunan, China; ^5^ Department of Gynecology, The Second Affiliated Hospital, Hengyang Medical School, University of South China, Hengyang, Hunan, China

**Keywords:** recurrent implantation failure, endometrial microbiota, endometrial tissue, major differential microorganisms, 16S ribosomal RNA gene sequencing

## Abstract

**Introduction:**

Recurrent implantation failure (RIF) is a major challenge in reproductive medicine, and emerging evidence suggests that alterations in the endometrial microbiota may play a critical role in its pathogenesis. To identify the main differential endometrial microbiota associated with RIF and to establish threshold values for their relative abundance.

**Methods:**

This case-control study was conducted in the Departments of Obstetrics, Gynecology, and Reproductive Medicine at two large hospitals. From March to December 2023, the study recruited 17 infertile patients with RIF (Group A, cases), 19 healthy non-pregnant women (Group B, controls), and 20 healthy pregnant women (Group C, controls). Demographic information, medication history, clinical data, and endometrial tissue samples were collected. Endometrial microbiota of all participants was profiled using 16S rRNA gene sequencing.

**Results:**

The richness of endometrial microbiota in Group A was significantly lower compared to both control groups (*P*=0.013, *P*=0.022, respectively). The diversity of endometrial microbiota in Group A and B was significantly higher compared to Group C (*P*=0.043, *P*=0.002, respectively). The composition of endometrial microbiota in Group A differed significantly from both control groups, whereas only minor differences were observed between the two control groups (PERMANOVA, *P*=0.001). *Methyloversatilis*, *Lactobacillus*, *Sphingomonas*, *Faecalibacterium*, *Escherichia-Shigella*, *Bacteroides*, and *Aeromonas* were identified as the main differential endometrial microbiota associated with RIF, with threshold relative abundances of 3.807%, 6.606%, 0.192%, 0.193% , 0.618%, 2.411%, and 0.019%, respectively. In Group A, *Lactobacillus* was positively correlated with *Sphingomonas* (*r*=0.64, *P*=0.005).

**Discussion:**

*Methyloversatilis*, *Lactobacillus*, *Sphingomonas*, *Faecalibacterium*, *Escherichia-Shigella*, *Bacteroides*, and *Aeromonas* were the main differential endometrial microbes associated with RIF. Preliminary threshold values for their relative abundances were established.

## Introduction

Recurrent implantation failure (RIF) presents a significant challenge in the field of reproductive medicine and remains a key barrier to successful outcomes in assisted reproductive technology. Current data indicated that 50%~60% of *in vitro* fertilization (IVF) cycles failed due to unsuccessful embryo implantation, with approximately 10% of cases classified as RIF (1–4). The etiology of RIF is complex, encompassing known factors such as anatomical and chromosomal abnormalities, impaired endometrial receptivity, immunological dysregulation, uterine pathologies, etc., as well as other unexplained causes, which bring great stress and frustration to both couples experiencing RIF and clinicians (5). The endometrium is a key tissue during the implantation of blastocysts, and the endometrial microbiota is crucial to the impact of RIF (6).

Female reproductive tract, such as the endometrium, is recognized as a polymicrobial niche where pathogenic and probiotic microbes coexist (1, 7). Increasing evidence suggested that endometrial microbiota played a significant role in RIF (8–10). To date, nearly all RIF studies have used IVF patients undergoing assisted reproductive technology as control groups. Given ongoing debate over whether the endometrial microbiota represents a promising target or an overestimated factor in reproductive outcomes, the value and implications of this study design merit further consideration (11). Perhaps we are seeking a molecular marker to predict treatment outcomes in RIF, or aiming to identify dysregulated endometrial microbial patterns to tailor personalized therapeutic strategies—or perhaps both. However, one key point has been inadvertently overlooked by all of us. Related studies have consistently demonstrated that the endometrial microbiota in infertile women is already dysregulated and abnormal (12–14). Therefore, to better translate endometrial microbiota research into clinical practice, comparisons should be made between RIF patients and reproductively healthy women.

This is the first study to compare the endometrial microbiota of RIF patients with that of healthy non-pregnant and pregnant women, aiming to identify significant differential microbiota. During the same study period, we established two control groups: one comprising healthy non-pregnant women at the time of endometrial tissue collection, and the other comprising women who conceived spontaneously and had successful implantation. The present study provides stronger evidence for investigating the role of endometrial microbiota in RIF patients and contributes to bridging the existing knowledge gap in translating molecular findings into clinical applications.

## Materials and methods

### Study design/study population

This was a case-control study conducted at the Department of Obstetrics, Gynecology, and Reproductive Medicine in the two large affiliated hospitals of the University of South China. The present study was approved by the Clinical Research Ethics Committee of the Second Hospital, University of South China ([2023]003, February 27, 2023). All participants willingly participated in the study and gave their written informed consent. We compared the microbiota composition of the endometrium among patients with RIF (Group A, n=17), healthy non-pregnant women (Group B, n=19), and healthy pregnant women who conceived spontaneously and had successful embryo implantation (Group C, n=20) from March 2023 to December 2023. Cases (Group A) and controls (Group B and C) were strictly identified following the established screening procedures, at an approximate ratio of 1:1:1. Special consideration was given to age to ensure that the age distribution in the case group was matched to those of the two control groups. Statistical analysis showed that there was no significant difference in age among the three groups.

Cases included only RIF patients (Group A), who were defined as women under 40 years old and had undergone 3 or more fresh or frozen embryo transfer cycles with a cumulative transfer of at least four high-quality embryos but had failed to achieve a clinical pregnancy (6, 15). Control groups consisted of Groups B and C. Group B comprised healthy women who were not pregnant at the time of endometrial sample collection. Group C included women in early pregnancy (≤9 weeks of gestation) who conceived spontaneously and chose to terminate the pregnancy through artificial abortion following successful embryo implantation.

Exclusion criteria mainly included women with ①abnormal vaginal bleeding; ② abnormal uterine morphology or pathology (such as uterine fibroids, severe pelvic adhesions, endometrial polyps, endometrial cancer, abnormal endometrial hyperplasia, endometriosis, adenomyosis, etc.); ③placed an intrauterine device, untreated hydrosalpinx, or abnormal sex hormone levels; ④acute inflammation, autoimmune diseases, endocrine diseases, sexually transmitted diseases, cancer, severe heart, brain, liver, or kidney diseases; ⑤any antibiotic, hormone, or immunosuppressive treatments within one month; ⑥cervical treatment within one week; ⑦vaginal douche or local vaginal medication within five days; or ⑧sexual intercourse within 48 hours.

### Data collection

The collected data include demographic characteristics (age, BMI, education level, household income), medication utilization evaluation, reproductive history, sexual behavior, number of pregnancies, deliveries, and abortions, as well as history of pelvic surgery. The cases underwent infertility-related examinations, including hysteroscopy at the time of sampling, RNA-seq-based endometrial receptivity analysis, gynecological examination, laboratory testing, imaging studies, and other assessments relevant to assisted reproductive technology treatment.

### Endometrial sampling

Endometrial specimens in Group A were collected using an endometrial sampler during RNA-seq-based endometrial receptivity testing, typically on the 5th day of progesterone-induced histological transformation in hormonally controlled cycles. Endometrial specimens in Group B were collected using an endometrial sampler on the 7th day after the surge of luteinizing hormone during the natural menstrual cycle (16). Endometrial specimens in Group C were collected using a disposable negative- pressure suction probe under B-ultrasound guidance, prior to the implementation of induced abortion surgery, to avoid contamination with embryonic villi. During ultrasound examination, accurate differentiation between the endometrium and the fetus can be achieved through careful assessment of their anatomical position, echogenic features, and gestational age, even when their images overlap or are difficult to distinguish.

High-resolution ultrasound equipment facilitated the acquisition of clearer images, while experienced physicians ensured precise sampling through comprehensive analysis of these image features. One endometrial tissue sample from each participant was used for the detection of chronic endometritis(CE). The diagnostic criteria for CE were defined as the presence of ≥5 CD138-positive plasma cells per 10–30 high-power field(HPF) based on immunohistochemical staining (17). The remaining endometrial tissue samples were rinsed with sterile saline and subsequently transferred into disposable sterile cryopreservation tubes (NEST, 607001) for storage at -80°C.

Certain measures were implemented to reduce the risk of specimen contamination. 1) During the study period, we assigned designated personnel for specimen collection, and the collection process was standardized and consistent. 2) Specimen collection was performed in operating rooms under regular monitoring of environmental hygiene. The microbial community in the air and on the work surfaces of the operating rooms was regularly monitored. These results indicated that no pathogenic bacteria were detected, with a total colony count of 0 CFU/m^3^ and 0 CFU/cm^2^, respectively. 3) The experimental procedure incorporated nuclease-free water as negative controls, which were processed in parallel with clinical specimens throughout the entire workflow, including DNA extraction, PCR amplification, library preparation, and sequencing. 4) The items utilized for specimen collection were sterilized. 5) Specimen collectors wore sterile gowns and sterile disposable gloves. 6) During the period, microbial communities on the hands of sample collectors and in disinfectants used on the skin or m (18)ucous membranes, such as 0.5% povidone iodine, were regularly tested. The results revealed no detection of pathogenic bacteria, with a total colony count of 0 CFU/cm² and 0 CFU/mL, respectively. 7) Specimens were collected prior to any treatment procedures.

### Endometrial microbiota testing

Endometrial microbiota was tested using 16S rRNA gene amplicon sequencing technology. Following thawing, endometrial tissue samples were vigorously vortexed in 2 mL of PBS(pH 7.0) to ensure homogeneous resuspension. The suspensions were then centrifuged at 12,000 rpm for 5 minutes, and the supernatants were discarded.

The remaining sediment were used for genomic DNA extraction using the CTAB method (Nobleryder, China). DNA quality was semi-quantitatively assessed by agarose gel electrophoresis, comparing the brightness of sample bands to that of a known DNA marker to estimate concentration and purity. DNA samples were diluted with sterile water to a concentration of 1ng/μL and then amplified by PCR. Secondly, the extracted DNA and region-specific primers (341F-806R) attached to Illumina paired-end (PE) adapters, barcodes, and sequencing primers were used to amplify the hypervariable V3-V4 region of 16S rRNA. The PCR products were purified using Qiagen Gel Extraction Kit (Qiagen, Germany) and subsequently assessed for their quality via electrophoresis on a 2% agarose gel. Finally, sequencing libraries were constructed using NEBNext^®^Ultra™II DNA Library Prep Kit, Cat No. E7645, and their quality was subsequently evaluated using a Qubit@ 2.0 Fluorometer (Thermo Scientific) and an Agilent Bioanalyzer 2100 System. Libraries were sequenced on the NovaSeq6000 platform (Illumina, USA) and 250 bp PE reads were created.

### Endometrial microbiota data analysis

Sequencing data was processed using the Quantitative Insights Into Microbial Ecology 2 (QIIME2) platform (https://qiime2.org/). High-quality clean reads were obtained through splicing the reads using FLASH software (V1.2.11) and controlling their quality using fastp software (V0.20.0). Chimeric sequences were detected and removed by comparing clean reads with the Silva database (https://www.arb-silva.de/) using Vsearch (V2.15.0). Amplicon sequence variants (ASVs) were generated by denoising effective reads using either the DADA2 or Deblur module in QIIME2 software (V QIIME2-202006). The Silva138.1 database was used to generate an ASVs table with taxonomy. ASVs with an abundance of less than 5 were excluded from the following analysis. The relative abundance of each ASV was standardized to the total abundance of all ASVs in each sample.

The microbial analysis platform was used for data analysis (Wekemo Bioincloud, https://www.bioincloud.tech). Alpha and beta diversity of the endometrial microbiota were assessed using Shannon diversity index, Chao index, principal coordinate analysis (PCoA), and permutational multivariate ANOVA (PERMANOVA) in R statistical software (v4.2.1, vegan package). A linear discrimination analysis (LDA) was implemented using LEfSe (R, DESeq2 package, Kruskal Wallis H-tests, two-tailed *P*-value <0.05, LDA score >4.0) to identify biomarkers at the genus level, which can distinguish the difference of microbiota composition among the three groups. Redundancy analysis (RDA) (R, vegan package, PERMANOVA, 999 permutations, *P*<0.05) was applied to assess the effects of host factors on microbial community. Microbial co-occurrence networks based on Spearman rank correlation (R, igraph package, Bray-Curtis, complete linkage; SPSS 26.0) was used to analyze the interactions between the microbe, considering taxa with a significant Spearman correlation coefficient (Benjamini-Hochberg corrected *P*<0.05). Statistical Analysis of Metagenomic Profiles (STAMP) software (V 2.1.3) was utilized to analyse the differences in mean proportions among microbial genera. Extended error bar plots (Welch’s t test or Kruskal-Wallis H test, two-tailed *P*-value <0.05, Benjamini-Hochberg multiple hypothesis test correction) displayed the microbial taxa exhibiting significant differences.

### Other data analysis

Continuous variables were described using means and standard deviation (SD) for data with normal distributions, or using medians and interquartile ranges for data with skewed distributions. Qualitative variables were reported using absolute frequencies and percentages. Intergroup differences were tested using a range of statistical tests, including Student’s t-test, Wilcoxon rank sum test, Kruskal-Wallis H test, chi-square test, or Fisher’s exact test. The relative abundance of the main endometrial microbiota associated with RIF was displayed using means and 95% confidence intervals in the three groups. This approach facilitates establishing a threshold range of the relative abundance for the main microbial community that affects recurrent implantation failure. A *P* value of <0.05 was considered statistically significant. Redundancy analysis (RDA) (Python 3.8, statsmodels and scikit-learn packages, PERMANOVA with 999 permutations, *P*<0.05) was performed to evaluate the associations between host factors and endometrial microbes. The relationships between the relative abundance of microbial genera and host factors were examined using linear regression models based on restricted cubic splines (RCSs) in Python (v3.8, statsmodels, patsy packages, *P* for nonlinearity < 0.05).

## Results

### Participants characteristics

A total of 56 participants were included in this study. Among the participants in Group A, all seventeen individuals had CE, with a prevalence rate of 100%. No cases of CE were observed in either Group B or Group C. **Table 1** presents a comprehensive report on the demographic characteristics of the study participants. No statistically significant differences were observed among the three groups in terms of key indicators such as age, body mass index, educational level, and family income. However, it is noteworthy that there were significant statistical differences in sexual and reproductive health-related indicators among the three groups. Specifically, Group A exhibited a significantly lower number of deliveries and induced abortions, as 12 women (70.6%) reported being nulliparous and 13 women (76.5%) reported no history of induced abortion. Compared to two control groups, only 1 woman (5.9%) reported engaging in ≥3 sexual activities per week, and 5 women had first intercourse ≤20 years old in Group A.

**Table 1:** Demographic characteristics of participants enrolled in the study.

Characteristics	Group A (n = 17)	Group B (n = 19)	Group C (n =20)	*P* value
Age, years, ± SD	31.76 ± 5.15	33.47 ± 5.20	31.55 ± 3.19	0.370^a^
Body mass index, kg/m^2^, ± SD	22.20 ± 3.29	22.24 ± 2.51	20.82 ± 2.82	0.229^a^
Educational level, n (%)				0.466^b^
Senior high school or lower	7 (41.18)	11 ( 57.89)	8 (40.00)	
Junior college or higher	10 (58.82)	8 (42.11)	12 (60.00)	
Household income, CNY / month, n (%)				0.097^b^
<5000	11 (64.71)	10 (52.63)	6 (30.00)	
≥5001	6 (35.29)	9 (47.37)	14 (70.00)	
Age of first sexual intercourse, years, n (%)				0.025^b^
≤20	5 (29.41)	14 (73.68)	9 (45.00)	
>20	12 (70.59)	5 (26.32)	11 (55.00)	
Frequency of sexual activity, /week, n (%)				0.012^c^
<3	16 (94.12)	17 (89.47)	11 (55.00)	
≥3	1 (5.88)	2 (10.53)	9 (45.00)	
Number of pregnancies, n (%)				0.003^b^
<4	14 (82.35)	13 (68.42)	7 (35.00)	
≥4	3 (17.65)	6 (31.58)	13 (65.00)	
Number of deliveries, n (%)				0.001^b^
0	12 (70.59)	7 (36.84)	2 (10.00)	
≥1	5 (29.41)	12 (63.16)	18 (90.00)	
Number of induced abortions, n (%)				0.042^b^
<2	13 (76.47)	10 (52.63)	7 (35.00)	
≥2	4 (23.53)	9 (47.37)	13 (65.00)	
History of pelvic surgery, n (%)				0.021^c^
Yes	1 (5.88)	4 (21.05)	9 (45.00)	
No	16 (94.12)	15 (78.95)	11 (55.00)	
Chronic endometritis, n (%)				<0.001^b^
Yes	17 (100.00)	0 (0.00)	0 (0.00)	
No	0 (0.00)	19 (100.00)	20 (100.00)	

Data are expressed as mean ± SD or absolute numbers and percentages.

Group A, patients with recurrent implantation failure; Group B, healthy women; Group C, healthy early pregnant women, who conceived spontaneously and had successful embryo implantation.

^a^Analysis of Variance; ^b^Chi-square test; ^c^Fisher’s exact test.

### Main differential endometrial microbiota associated with RIF

**Figure 1:**
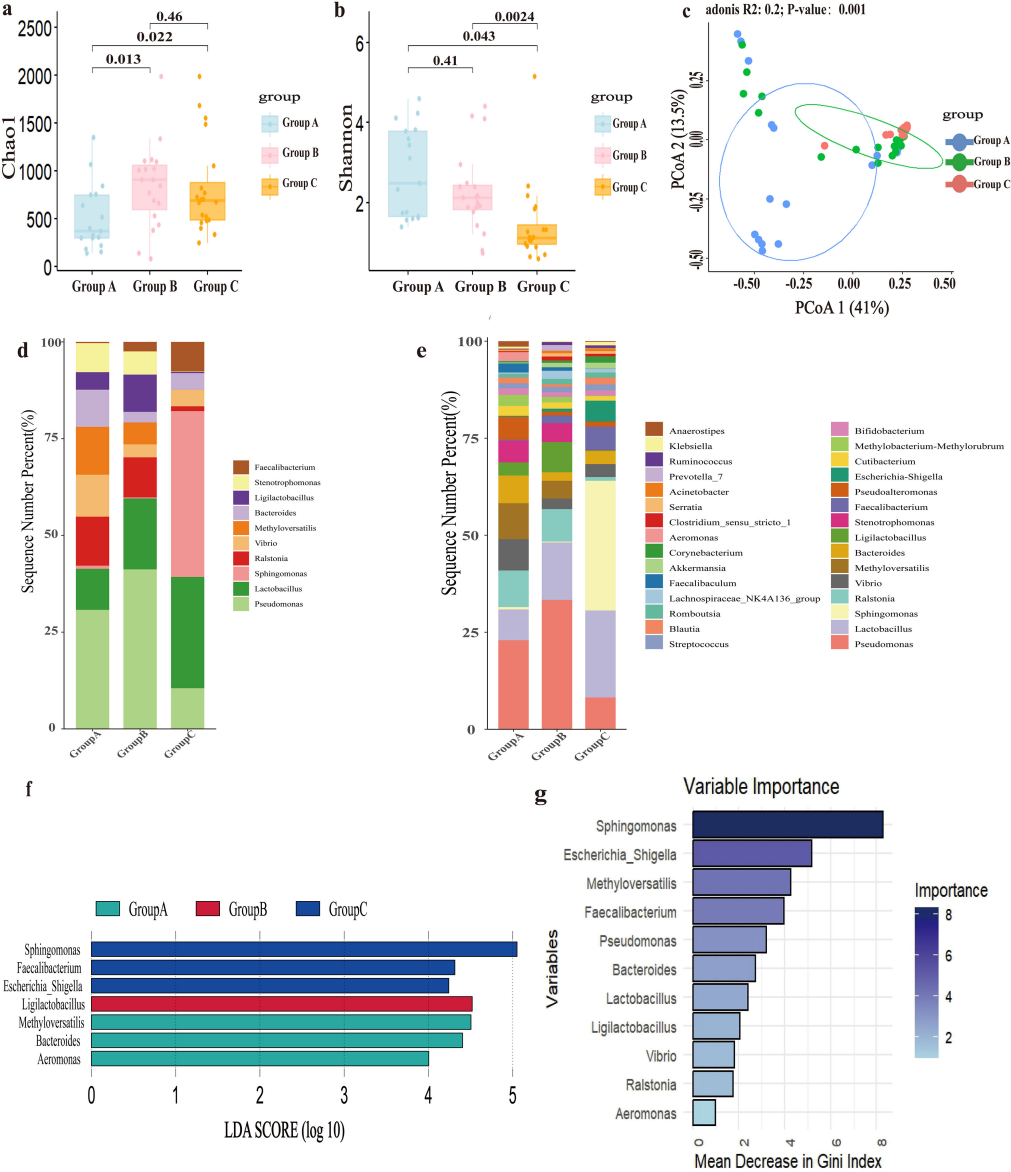
The composition and differences of endometrial microbiota. Group A, patients with recurrent implantation failure; Group B, healthy women; Group C, healthy early pregnant women, who conceived spontaneously and had successful embryo implantation. **(a)** Richness of endometrial microbiota, Chao index. **(b)** Diversity of endometrial microbiota, Shannon’s index. **(c)** Principal Co-ordinates Analysis (PCoA) based on Bray-Curtis distance calculation. **(d)** Top 10 microbial genera relative abundance bar chart. **(e)** Top 30 microbial genera relative abundance bar chart. **(f)** Bar chart of LDA value distribution (set value: LDA score>4). **(g)** Random forest model.

Diversity of endometrial microbiota was analyzed in **Figures 1a-c**. The richness of the endometrial microbiota in Group A was significantly lower than that in both control groups (**Figure 1a**). There were no statistically significant differences between Group B and Group C. Based on Shannon’s diversity index, Group A and B exhibited significantly higher microbial diversity than Group C (**Figure 1b**). **Figure 1c** demonstrated that endometrial microbial composition of Group A differed significantly from those of the other two control groups, while only minor differences were observed between the two control groups. RIF explained 20% of the variations in the composition of endometrial microbiota (PERMANOVA, *P*=0.001).

Differential analysis of the endometrial microbiota composition was conducted, with the results presented in **Figures 1d, e**. Among the three groups, the relative abundance of *Lactobacillus* showed the most significant changes, with the lowest relative abundance in Group A (6.6%) and the highest relative abundance in Group C (16.1%). *Pseudomonas*, as the top genus, had the highest relative abundance in Group B (27.7%), followed by Group A (19.1%), and finally Group C (5.9%). Compared to the two control groups, *Ralstonia* (7.9%), *Methyloversatilis* (7.7%), *Vibrio* (6.7%), *Bacteroides* (6.0%), *Pseudoalteromonas* (5.0%), *Stenotrophomonas* (4.7%), *Methylobacterium* (2.4%), *Cutibacterium* (2.1%), *Sphingomonas* (0.5%), *Escherichia_Shigella* (0.2%), and *Faecalibacterium* (0.2%) had significant relative abundance changes in Group A. According to the criteria of LDA score greater than 4, *Methyloversatilis*, *Bacteroides* and *Aeromonas* were the main differential microbiota in Group A; *Ligilactobacillus* was the main differential microbiota in Group B; and *Sphingomonas*, *Faecalibacterium*, and *Escherichia_Shigella* were the main differential microbiota in Group C (**Figure 1f**).

This study employed a random forest model to further identify main differential endometrial microbes among the three groups. *Sphingomonas*, *Escherichia-Shigella*, *Methyloversatilis*, and *Faecalibacterium* were the four most important genera with relatively high Gini coefficients (**Figure 1g**).

In light of the above results, *Methyloversatilis*, *Lactobacillus*, *Sphingomonas*, *Faecalibacterium*, *Escherichia-Shigella*, *Bacteroides*, and *Aeromonas* were identified as the main differential endometrial microbes associated with RIF. It should be emphasized that *Lactobacillus* not only represented the second major genus but also exhibited the most pronounced variations in relative abundance among the three groups.

### The threshold values for the relative abundance of the main differential microbes associated with RIF

In this study, the mean and 95% confidence intervals (CI) of the relative abundance for the main differential microbiota in each group were calculated to estimate their threshold values associated with RIF (**Table 2**). Differential microbial relative abundance thresholds was defined based on functional characteristics. For embryo implantation-disrupting microbes (e.g., *Methyloversatilis*, *Aeromonas*), the higher mean value between the healthy non-pregnant group (Group B) and the early pregnancy group (Group C) was selected as the pathogenic threshold. For embryo implantation-promoting microbes (e.g., *Lactobacillus*), the mean value in the RIF group (Group A, 6.606%) was adopted as the protective threshold. This strategy ensured statistically significant differences (*P*<0.05) by comparing 95% confidence intervals(**Table 2**). The threshold values for relative abundance were determined as follows: 3.807% for *Methyloversatilis*, 6.606% for *Lactobacillus*, 0.192% for *Sphingomonas*, 0.193% for *Faecalibacterium*, 0.618% for *Escherichia-Shigella*, 2.411% for *Bacteroides*, and 0.019% for *Aeromonas.* In Group C, *Methyloversatilis* was undetectable in 19 individuals and detected at a low level (0.045%) in only one individual. Therefore, the risk of RIF increased when the relative abundance of *Methyloversatilis, Bacteroides*, *Sphingomonas*, *and Aeromonas* exceeded their respective thresholds, or when the relative abundance of *Lactobacillus*, *Faecalibacterium*, or *Escherichia-Shigella* fell below the defined threshold values.

**Table 2:** Mean and 95% CI of Relative Abundance for Main Differential Endometrial Microbiota in Each Group.

Differential microbiota	Group A	Group B	Group C
*Methyloversatilis*, %	7.705 (1.979-13.431)	3.807 (2.421-5.192)	0.006 (0-0.015)
*Lactobacillus*, %	6.606 (0.754-12.458)	12.298 (2.181-22.415)	16.127 (4.985-27.269)
*Sphingomonas*, %	0.504 (0-1.347)	0.192 (0.085-0.299)	24.039 (13.592-34.485)
*Faecalibacterium*, %	0.193 (0-0.406)	1.164 (0.374-2.904)	4.289 (2.174-6.405)
*Escherichia-Shigella*, %	0.191 (0-0.394)	0.618 (0.396-0.840)	3.876 (0.218-7.533)
*Bacteroides*, %	5.968 (0-14.446)	1.838 (0.552-3.124)	2.411 (0.277-4.546)
*Aeromonas*, %	1.938 (0.347-3.529)	0.019 (0.004-0.034)	0.009 (0-0.023)

Data are expressed as mean and 95% confidence interval.

Group A, patients with recurrent implantation failure; Group B, healthy women; Group C, healthy early pregnant women, who conceived spontaneously and had successful embryo implantation.

### Microbial taxa interactions

**Figure 2:**
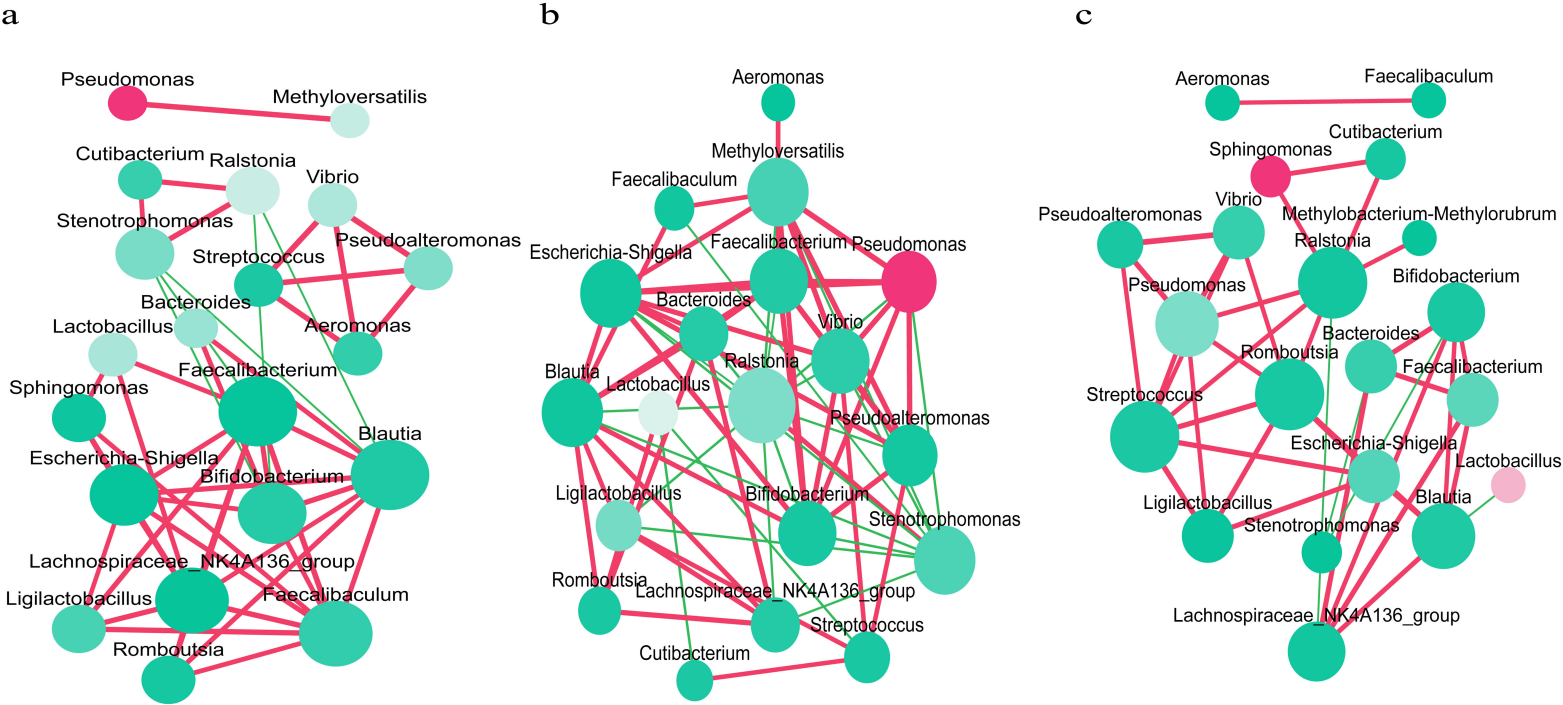
Interaction networks of the endometrial microbiota in each group. **(a)** Interaction network analysis of the endometrial microbiota in recurrent implantation failure patients group. **(b)** Interaction network analysis of the endometrial microbiota in healthy women group. **(c)** Interaction network analysis of the endometrial microbiota in healthy early pregnant women group, who conceived spontaneously and had successful embryo implantation. Each microbe is represented by a node, and the size of the node indicates the number of connections that microbe has with other microbes. The larger the number of connections, the larger the size of the node. The color of the node indicates the relative abundance of microbes, with a redder color indicating a greater relative abundance. The connecting lines represent the degree of correlation, with red indicating a positive correlation and green indicating a negative correlation. The deeper the color of the line, the stronger the correlation (*P*<0.05 after *Benjamini-Hochberg* correction).

Interaction networks of endometrial microbiota were established for each group (**Figure 2**). In Group A, *Lactobacillus* was positively correlated with *Sphingomonas* (*r=* 0.64, *P=* 0.005; **Figure 2a**), indicating an enhanced synergistic interaction between *Lactobacillus* and *Sphingomonas*. There was a significant positive correlation between *Pseudomonas* and *Methyloversatilis* (r=0.86, *P*<0.001; **Figure 2a**). In Group B, *Lactobacillus* exhibited a negative correlation with *Cutibacterium* and *Streptococcus* (r=-0.49, *P=*0.033; r=-0.53, *P*=0.018; **Figure 2b**). *Pseudomonas* displayed a significant positive correlation with *Vibrio*, *Methyloversatilis*, *Pseudoalteromonas, Faecalibacterium*, and *Escherichia-Shigella* (r=0.81, *P*<0.001; r=0.93, *P*<0.001; r=0.83, *P*<0.001; r= 0.64, *P=*0.003; r=0.65, *P=*0.003; **Figure 2b**). *Aeromonas* and *Methyloversatilis* exhibited a positive correlation (r=0.52, *P=*0.022; **Figure 2b**). In Group C, *Pseudomonas* exhibited a significant positive correlation with *Ralstonia*, *Vibrio*, and *Pseudoalteromonas* (r=0.62, *P=*0.004; r=0.83, *P*<0.001; r=0.77, *P*<0.001; **Figure 2c**).

### Associations between endometrial microbiota and host factors

Associations between host factors and the relative abundance of the top 30 microbial genera are presented in **Supplementary Table 1**. Statistically significant associations with endometrial microbiota composition were observed for the number of induced abortions (*R^2^ =* 7.09%, *P*=0.002), pregnancies (*R^2^ =* 5.22%, *P*=0.003), deliveries (*R^2^ =* 5.22%, *P*=0.006), and frequency of sexual activity (*R^2^ =* 4.10%, *P*= 0.022). History of pelvic surgery(*R^2^ =* 2.91%, *P*=0.108) and age at first sexual intercourse(*R^2^ =* 2.26%, *P*=0.242) were not significantly associated with endometrial microbiota composition.

**Figure 3:**
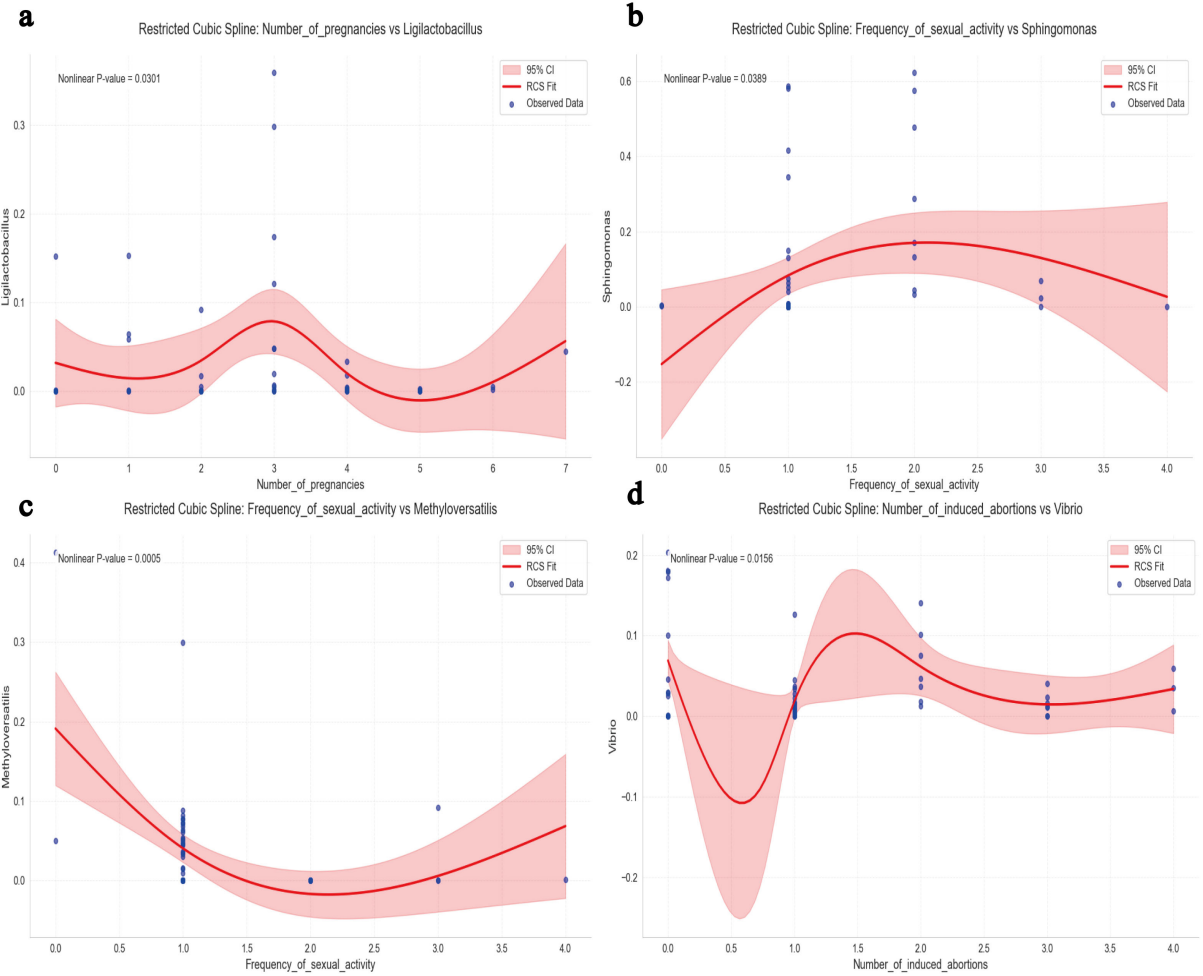
Multivariable restricted cubic spline (RCS) joint analysis. **(a)** RCS Analysis of *Ligilactobacillus* Relative Abundance in Relation to the Number of Pregnancies. **(b)** RCS Analysis of *Sphingomonas* Relative Abundance in Relation to Frequency of Sexual Activity. **(c)** RCS Analysis of *Methyloversatilis* Relative Abundance in Relation to Frequency of Sexual Activity. **(d)** RCS Analysis of *Vibrio* Relative Abundance in Relation to the Number of Induced Abortions. A two-tailed *P* value for non-linearity <0.05 was considered statistically significant. The red curve represents the spline-fitted trend, and the shaded area denotes the 95% confidence interval(CI).

As shown in **Figure 3a**, restricted cubic spline analysis revealed the non-linear relationship between the number of pregnancies and the relative abundance of *Ligilactobacillus*(*P*=0.0301). The relative abundance peaked at three pregnancies, declined with both fewer and more pregnancies, and exhibited an increase when the number exceeded five. A significant inverse U-shaped association was observed between the frequency of sexual activity and the relative abundance of *Sphingomonas*(**Figure 3b**, *P*=0.0389). Abundance increased with frequency up to around two times per week, then declined thereafter, indicating a threshold effect in the microbial response. Conversely, a significant U-shaped association was identified between the frequency of sexual activity and the relative abundance of *Methyloversatilis* (**Figure 3c**, *P*=0.0005). The abundance declined with increasing frequency up to around two times per week, after which it rose sharply, indicating a non-linear microbial response to behavioral exposure. The number of induced abortions had a significant non-linear relationship with the relative abundance of *Vibrio* (**Figure 3d**, *P*=0.0156). *Vibrio* abundance was lowest at one induced abortion and increased steadily with a higher number of abortions. This pattern suggested a dose-dependent microbial response potentially linked to cumulative reproductive tract disturbance.

## Discussion

### Main findings

In the present study, there were significant differences in the composition of endometrial microbiota between RIF patients and both healthy non-pregnant women and healthy pregnant women. *Methyloversatilis*, *Lactobacillus*, *Sphingomonas*, *Faecalibacterium*, *Escherichia-Shigella*, *Bacteroides*, and *Aeromonas* were identified as the main differential endometrial microbes associated with RIF. Preliminary reference thresholds for their relative abundance were defined. These differential microbiota exhibited different interactions at different relative abundances.

### Interpretation of results

Our findings further supported that endometrial microbiota was closely associated with the occurrence of RIF (6, 10, 19). Previous studies have shown that the composition of endometrial microbiota in RIF is significantly different from that of other women undergoing assisted reproductive technology treatment (6, 20–22). Based on these studies, this study’s design may help to clarify a potential pathophysiological mechanism linking endometrial microbiota and RIF (10). Compared to healthy women with normal fertility, the endometrial microbiota of RIF patients exhibited reduced richness but increased diversity. Principal Coordinates Analysis (PCoA) further revealed distinct microbial community structures, highlighting the presence of numerous differential taxa in the endometrium of RIF patients. We identified *Methyloversatilis*, *Lactobacillus*, *Sphingomonas*, *Faecalibacterium*, *Escherichia-Shigella*, *Bacteroides*, and *Aeromonas* as the main differential endometrial microbes associated with RIF. Among them, *Lactobacillus*, *Sphingomonas*, and *Faecalibacterium* appeared to be favorable for pregnancy in women (23, 24). *Lactobacillus* can impact the function of the endometrium by influencing its immune system, receptivity, and remodeling, thereby affecting female pregnancy (25–27). The effects of *Sphingomonas* on the endometrium remain unclear. However, a study found that it could inhibit the infection of cervical HPV and the occurrence of cervical cancer (28). *Faecalibacterium* was a beneficial genus for human health (29). When its relative abundance was high, it had a certain anti-inflammatory effect; however, when its abundance was low, it could easily lead to mucosal inflammation (29–31). *Methyloversatilis* and *Pseudomonas* were pathogenic bacteria that contribute to the occurrence of RIF. *Methyloversatilis* had been found in the female reproductive tract (32), and its effect on the endometrium was currently unclear. Currently, it was believed that *Pseudomonas* was an opportunistic pathogen in human urogenital system (28). Genomic versatility of *Pseudomonas* enabled it to encode and produced a diverse array of cell-associated and extracellular virulence factors, thereby facilitating its successful colonization in various host environments and contributing significantly to its elevated levels of antibiotic resistance (33). This factor might pose a challenge in RIF treatment. By comparing the endometrial microbiota of healthy women with normal fertility, we established threshold values for the main differential microbiota for the first time. In the endometrial microenvironment of RIF, there existed a dynamic antagonistic interaction between *Lactobacillus* and *Pseudomonas*. In a healthy endometrial environment, *Lactobacillus* dominates by maintaining acidity, secreting antimicrobial substances, and competitively occupying ecological niches, thereby suppressing the growth of *Pseudomonas* and limiting it to low-abundance, benign colonization (23, 25). However, in the pathological state of RIF, the abundance of *Lactobacillus* decreased significantly, compromising the microbiota barrier. Its loss of dominance lifted suppression on *Pseudomonas* and might facilitate the overgrowth of other microbes, further disrupting the endometrial microenvironment (34). Notably, a decline in *Lactobacillus* was accompanied by an increase in microbes like *Methyloversatilis*, which may synergize with *Pseudomonas*. The increased abundance of *Pseudomonas* has been associated with inflammatory responses and adverse reproductive outcomes, suggesting its potential transition into a highly virulent pathogen (34). This association indicated that *Lactobacillus* depletion might be a key trigger for *Pseudomonas* virulence activation, creating a vicious cycle of “dysbiosis-inflammation-gene suppression”. Importantly, disruption of the endometrial microbiota resulted in marked shifts in community composition and inter-microbial interactions, including the enhanced synergy between *Pseudomonas* and *Methyloversatilis*. Such ecological changes might further compromise the integrity of the endometrial microenvironment.

This study has uncovered a significant correlation between RIF, chronic endometritis (CE) and specific microbial communities. Existing research has demonstrated that CE is closely associated with marked alterations in the endometrial microbiota, which may disrupt the endometrial immune environment and consequently influence the success rate of embryo implantation (35). Some evidence indicated that microbial dysbiosis in CE patients, especially the increased presence of non-*Lactobacillus* microbes, may be associated with endometrial inflammation (18, 22, 34). CE may create a favorable microenvironment for the proliferation of certain pathogenic microbes, potentially contributing to dysbiosis of the endometrial microbiota (18). Further research is needed to fully elucidate the underlying mechanisms of these associations.

Our study suggested that host factors, including induced abortions, number of pregnancies, and frequency of sexual activity, significantly influence the composition of the endometrial microbiota. The relative abundance of *Ligilactobacillus* peaked in women with three pregnancies, decreased thereafter, and rebounded after five pregnancies, suggesting a compensatory microbial response to physiological stress. This pattern indicated that the microbiota might adapt to stress through compensatory mechanisms, highlighting the adaptive plasticity of the probiotic ecosystem under pressure (35). Notably, when sexual activity exceeded twice per week, the abundance of *Sphingomonas* decreased while *Methyloversatilis* increased, indicating that frequent sexual activity might disrupt the microbial barrier and promote colonization by opportunistic pathogens (36, 37). Additionally, a higher number of induced abortions was positively correlated with increased *Vibrio* abundance, implying that repeated abortions might exacerbate inflammation and promote pathogen proliferation (38). These findings underscored the role of reproductive factors as modifiable drivers of endometrial dysbiosis and highlighted critical thresholds, such as three pregnancies and twice-weekly sexual activity, that may serve as early intervention targets.

Notably, this study observed a high prevalence of CE at 30.4%, suggesting that CE was associated with the study results through two distinct mechanisms. First, as a key coexisting factor, endometrial microbiota can both cause CE and be affected by it (18, 34, 39). On the one hand, microbial infections may lead to the development of CE, thereby affecting embryo implantation; on the other hand, CE may alter the microbial composition through persistent inflammatory responses, thereby potentially confounding the true associations between host factors and the microbiota (18, 34). Second, as an effect modifier, the chronic inflammatory microenvironment of CE may potentially intensify the impact of reproductive history and sexual behavior on the microbiota by weakening mucosal defenses and shifting local immune tolerance thresholds (19, 22, 40).

### Study strengths

This study is the first to investigate and identify the main differential microbiota associated with RIF by comparing healthy non-pregnant women, healthy pregnant women, and RIF patients. These findings, including the identification of the main differential microbiota, the establishment of threshold values for their relative abundance, and the construction of their interaction networks, aim to advance the development of endometrial microbiota-based strategies for the intervention and treatment of RIF. The study’s objectives and scope align closely with global research priorities focused on the role of the female reproductive tract microbiota in reproductive health (23, 41–43).

### Limitations

This study has some limitations. Firstly, this study can not directly prove the causal relationship between endometrial microbiota and RIF. Although a strong correlation between them has been observed, the study’s design and methodological limitations preclude the establishment of a causal relationship between the microbiota and RIF or between the microbiota, CE, and RIF. It is challenging to collect endometrial tissue, detect endometrial microbiota, and follow up on whether RIF occurs in women of childbearing age who have not yet experienced any reproductive problems (which is an obstacle in any study). Secondly, this study was limited by a relatively small sample size. Several factors contributed to this constraint. The collection of endometrial tissue required sterile procedures performed by senior gynecologists under sterile conditions in a hospital setting. For the control groups, endometrial tissue had to be obtained from healthy women who met strict inclusion and exclusion criteria—criteria that most hospital visitors did not fulfill. For the case group, endometrial tissue was collected specifically during RNA-seq-based endometrial receptivity testing, in order to closely mimic the conditions of embryo transfer. Thirdly, while threshold values for the main differential microbiota were preliminarily established, further validation in larger, independent cohorts is needed. Lastly, although multiple confounding factors were controlled for in the study, the potential influence of CE as a confounder in the relationship between host factors and endometrial microbiota cannot be entirely ruled out. The high prevalence of CE may have impacted the observed associations between endometrial microbiota and host factors, potentially contributing to non-linear patterns or threshold effects. These effects may stem from the selective pressure exerted on the microbial community by the chronic inflammatory environment associated with CE. Given the potential impact of CE on endometrial microbiota, further investigation into the interaction between CE and host factors is essential for elucidating the underlying mechanisms of reproductive disorders.

### Future perspectives

This study could initiate several important next steps. First, it is essential to refine the threshold ranges of relative abundance for key differential endometrial microbiota and to assess their clinical predictive value in relation to reproductive outcomes, particularly recurrent implantation failure (RIF). Establishing standardized cutoff values may enhance the utility of microbial biomarkers in clinical risk stratification and early intervention. Second, mechanistic studies using animal models are warranted to validate the causal roles of these dominant microbial taxa and to explore their interactions within the endometrial microenvironment. These studies could elucidate the underlying pathways through which microbial dysbiosis contributes to implantation failure, thereby informing the development of targeted microbiota-based therapies or immunomodulatory strategies.

## Conclusions

*Methyloversatilis*, *Lactobacillus*, *Sphingomonas*, *Faecalibacterium*, *Escherichia-Shigella*, *Bacteroides*, and *Aeromonas* were identified as the key differential endometrial microbes associated with recurrent implantation failure (RIF). Preliminary threshold values for the relative abundance of these major taxa were established.

## Author's note

This study sheds light on the pivotal role of endometrial microbiota in RIF, an emerging challenge in assisted reproductive technology. As the first case-control study to compare the endometrial microbiota of RIF patients with both healthy nonpregnant and pregnant women, it aimed to identify significant differential microbiota and establish preliminary threshold values for their relative abundances. Significant differences in endometrial microbiota richness and diversity were observed between RIF patients and the control groups. *Methyloversatilis*, *Lactobacillus*, *Sphingomonas*, *Faecalibacterium*, *Escherichia-Shigella*, *Bacteroides*, and *Aeromonas* were identified as the main differential endometrial microbiota associated with RIF. Understanding the microbial dynamics of the endometrium is crucial for elucidating the underlying mechanisms of RIF and helps in developing novel diagnostic and therapeutic strategies. These findings hold significant implications for improving reproductive outcomes and enhancing patient care in reproductive medicine.

## Data Availability

The authors acknowledge that the data presented in this study must be deposited and made publicly available in an acceptable repository, prior to publication. Frontiers cannot accept an article that does not adhere to our open data policies.
