# Supplementary material for: Main differential endometrial microbiota associated with recurrent implantation failure: a case control study
Source: Front Endocrinol (Lausanne). 2025 Aug 11;16:1504609. doi: 10.3389/fendo.2025.1504609 (PMC12375461; doi:10.3389/fendo.2025.1504609)
Supplement: Supplementary file 2 [file Presentation1.pdf]

Research protocol

The primary aim of this study is to explore the impact of *Lactobacillus, Pseudomonas, Methyloversatilis*, and chronic endometritis on recurrent embryo implantation failure and to determine the threshold range of the abundance for these differential microbiota that affects chronic endometritis and recurrent embryo implantation failure.

1 Research Design

This project plans to use a prospective case-control study.

2 Study Population

Control group 1 will consist of women of normal fertility who are not pregnant, selected from two tertiary hospitals affiliated with the University of South China, Hunan, China. These women had previously experienced successful pregnancies or conceived spontaneously after enrollment, with normal fetal development. Control group 2 will comprise early pregnant women who conceived spontaneously (within 8^{+6}weeks, determined by the clinical time of surgical abortion) with normal embryonic development but opted for terminate the pregnancy through induced abortion. Case group will consist of patients with recurrent embryo implantation failure selected from the Reproductive Medicine Center.

Rationale for Selecting the Control Groups

This project focuses on the uterine microbiome, and the selection of the control groups was based on relevant research findings. Controls will be not selected from infertile women who achieved embryo implantation because numerous studies have reported significant differences in the uterine microbiome between infertile women and those with normal fertility, which is closely associated with infertility. Thus, to ensure the accuracy and reliability of the study results, this project chose women with normal fertility with or without pregnancy, as the control groups. Additionally, our previous research analyzed the uterine microbiome of both control groups, showing both similarities and differences. Hence, selecting women with normal fertility in two

different states (non-pregnant and successfully implanted) as controls is crucial, especially for determining the pathogenic threshold of differential microbiota.

Special Design of the Case Group

(1) The embryo implantation window will be defined for all study participants.

(2) Endometrial tissue collection will be performed on the 5th day of the artificially induced cycle for frozen-thawed embryo transfer, ensuring that hormone levels and endometrial receptivity during collection are consistent with those at the time of embryo transfer.

(3) All participants will be from the same reproductive medicine center, with consistent conditions for embryo culture, embryo transfer, technical personnel, procedures, infrastructure, equipment, culture media, and consumables. This design minimized confounding factors that could influence the endometrial microbiome, embryo quality, and pregnancy outcomes in the case group, thereby ensuring the accuracy and reliability of the research results.

Inclusion Criteria for the Case Group:

(1) Women aged 20–40 years.
(2) BMI between 18.5 and 23.9 kg/m^2.
(3) Women who have not conceived after the transfer of at least four high-quality embryos across three fresh or frozen cycles.
(4) Normal ovarian reserve (FSH $<$ 10 mIU/mL, AMH $>$ 1.1 ng/mL, AFC $>$ 7).
(5) Normal karyotypes for both partners.
(6) No infection detected in cervical and vaginal secretions within one week before endometrial receptivity testing.
(7) Signed informed consent, no cognitive impairment, and willing to cooperate with data and biological sample collection.

Exclusion Criteria for the Case Group:

(1) History of abnormal vaginal bleeding.
(2) Abnormal uterine morphology or pathology (e.g., submucosal fibroids, intrauterine adhesions, endometrial polyps, endometrial carcinoma, endometrial hyperplasia, endometriosis).

(3) Ovarian cancer, untreated hydrosalpinx, abnormal baseline hormone levels.
(4) Infectious diseases, autoimmune diseases, endocrine disorders, malignant tumors, tuberculosis, sexually transmitted diseases, severe cardiovascular, cerebrovascular, hepatic, or renal diseases.
(5) Use of antibiotics, hormones, or immunosuppressants within the past month.
(6) Cervical treatments within the last week.
(7) Vaginal douching or local medication within the past five days.
(8) Sexual activity within the past 48 hours.
(9) Male factor infertility (e.g., severe oligospermia, asthenospermia, obstructive azoospermia).

Inclusion Criteria for Control Group 1:

(1) Women aged 20–40 years.
(2) BMI between 18.5 and 23.9 kg/m^2.
(3) Natural conception and normal fetal development after enrollment, or having had children previously through natural conception.
(4) Signed informed consent, no cognitive impairment, and willing to cooperate with data and biological sample collection.

Inclusion Criteria for Control Group 2:

(1) Women aged 20–40 years.
(2) BMI between 18.5 and 23.9 kg/m^2.
(3) Natural conception.
(4) Early pregnancy with a confirmed live intrauterine embryo.
(5) Voluntarily choosing surgical abortion to terminate the pregnancy.
(6) Signed informed consent, no cognitive impairment, and willing to cooperate with data and biological sample collection.

Exclusion Criteria for Control Groups 1 and 2:

(1) History of abnormal vaginal bleeding or inflammation.
(2) Exclusion criteria 2–8 are consistent with those for the case group.

3 Sample Size Estimation

Based on literature reviews, the exposure rate of chronic endometritis caused by

differential microbiota in recurrent implantation failure is unclear. Therefore, this study calculates the sample size using an estimated low exposure rate of 28.6%. The expected odds ratio (OR) is 3.0, with $\alpha = 0.05$, $\beta = 0.10$. To ensure a sufficient sample size, a dropout rate of 20% was set, resulting in the final inclusion of 56 participants in the case group, control group 1, and control group 2.

4 Diagnostic Criteria for Key Indicators

(1) Chronic endometritis: Based on clinical guidelines and recent studies, endometrial tissue stained with immunohistochemistry showing positive expression of plasma cells CD138 and CD38. In one high-power field (HPF) with the highest CD138 expression, ≥5 plasma cells per HPF.

(2) Recurrent implantation failure: Women under 40 who fail to achieve clinical pregnancy after at least 4 high-quality embryo transfers across three cycles.

(3) Early pregnancy live fetus diagnosis: Missed menstruation, positive serum or urine β-HCG, and confirmation of gestational sac, embryo, and primitive cardiac activity by transvaginal Doppler ultrasound.

(4) Implantation window: A specific, short period when the endometrium allows embryo implantation, usually occurring on days 19–24 of the menstrual cycle, or on day 5 of progesterone administration in an artificial cycle.

(5) RNA-seq-based endometrial receptivity testing: Analysis of endometrial receptivity gene expression, predicting the current endometrial receptivity status (pre-receptive, receptive, post-receptive) to optimize embryo transfer timing and improve pregnancy rates.

(6) High-quality embryo criteria: Based on the Peter cleavage stage embryo scoring system (≥7 cells, ≤20% fragmentation on day 3 post-fertilization) and Gardner blastocyst scoring (≥3BB on days 5–6).

5 Data Collection

Our study will collect general demographic information, gynecological examinations, laboratory tests, imaging, hysteroscopic examinations, and data related to assisted reproductive technology treatments. Biological samples were also collected from the participants. Each participant was assigned a unique identifier that corresponded to all

their relevant data.

(1) Demographic Data: The demographic questionnaire primarily included basic demographic information, medical history, marital and reproductive history, sexual history, and menstrual cycle details. Physical examination data primarily included height and weight.

(2) Gynecological Examination Data: This included bimanual palpation, vaginal secretion analysis, cervical morphology examination, cervical TCT (thin-prep cytology test), and HPV testing.

(3) Laboratory Test Data: This included routine blood tests, erythrocyte sedimentation rate, coagulation function, C-reactive protein, six reproductive hormones, and blood/urine β-HCG.

(4) Imaging Data: This primarily included transvaginal color Doppler ultrasound results, such as uterine morphology (length, width, thickness, volume, etc.), endometrial receptivity (endometrial thickness, triple-line pattern, endometrial volume, uterine artery and endometrial hemodynamic parameters, subendometrial blood flow index, endometrial peristaltic wave patterns and frequency), and embryonic development.

(5) Hysteroscopic Examination Data: This included the shape of the cervical canal, uterine cavity, and tubal ostia.

(6) Embryo Transfer Cycle Data: This included the endometrial preparation protocol during frozen-thawed cycles, thawed embryo grading, number of thawed embryos, endometrial thickness and pattern on the day of endometrial transformation, estrogen and progesterone levels on the day of endometrial transformation and one day before embryo transfer, the timing of embryo transfer (i.e., the implantation window based on endometrial receptivity testing), and the outcome of the frozen-thawed cycle.

6 Collection of Biological Samples

Endometrial tissue specimens, amounting to approximately 0.5–1g in total, will be collected. One sample will be used for 16S rRNA gene sequencing to analyze microbial composition, another one for immunohistochemistry and pathological

analysis, and a third sample will be stored in an ultra-low-temperature freezer (-80°C) for future utilization.

7 Biological Sample Testing

Endometrial tissues will be collected for the following tests:

- Immunohistochemistry for CD138 and CD38 expression.
- 16S rRNA sequencing to identify the types and relative abundance of microbial communities in the endometrial tissue.
